# Mass-Spectrometric Evaluation of the African Swine Fever Virus-Induced Host Shutoff Using Dynamic Stable Isotope Labeling with Amino Acids in Cell Culture (SILAC)

**DOI:** 10.3390/v15061283

**Published:** 2023-05-30

**Authors:** Elisabeth Wöhnke, Barbara G. Klupp, Sandra Blome, Thomas C. Mettenleiter, Axel Karger

**Affiliations:** 1 Institute of Molecular Virology and Cell Biology, Friedrich-Loeffler-Institut, Federal Research Institute for Animal Health, Südufer 10, 17493 Greifswald-Insel Riems, Germany; 2 Institute of Diagnostic Virology, Friedrich-Loeffler-Institut, Federal Research Institute for Animal Health, Südufer 10, 17493 Greifswald-Insel Riems, Germany; 3 Friedrich-Loeffler-Institut, Federal Research Institute for Animal Health, Südufer 10, 17493 Greifswald-Insel Riems, Germany

**Keywords:** African swine fever virus, ASFV, virus-induced host shutoff, vhs, SILAC, PrV, pseudorabies virus, porcine macrophages, mass spectrometry, proteomics

## Abstract

African swine fever is a viral disease of swine caused by the African swine fever virus (ASFV). Currently, ASFV is spreading over the Eurasian continent and threatening global pig husbandry. One viral strategy to undermine an efficient host cell response is to establish a global shutoff of host protein synthesis. This shutoff has been observed in ASFV-infected cultured cells using two-dimensional electrophoresis combined with metabolic radioactive labeling. However, it remained unclear if this shutoff was selective for certain host proteins. Here, we characterized ASFV-induced shutoff in porcine macrophages by measurement of relative protein synthesis rates using a mass spectrometric approach based on stable isotope labeling with amino acids in cell culture (SILAC). The impact of ASFV infection on the synthesis of >2000 individual host proteins showed a high degree of variability, ranging from complete shutoff to a strong induction of proteins that are absent from naïve cells. GO-term enrichment analysis revealed that the most effective shutoff was observed for proteins related to RNA metabolism, while typical representatives of the innate immune system were strongly induced after infection. This experimental setup is suitable to quantify a virion-induced host shutoff (vhs) after infection with different viruses.

## 1. Introduction

Protein homeostasis is maintained by the well balanced control of the protein turnover, which is determined by the rates of protein synthesis, modification, and degradation. The turnover is adjusted to the cellular requirements at different stages of differentiation or under stress conditions, such as starvation, heat, or unphysiological oxygen pressure, treatment with chemical or biological stimulants, or infection. Many viruses, including herpesviruses, influenza viruses, and vaccinia virus (VV), induce a generalized shutoff of host cell protein synthesis (virus or virion-induced host shutoff, vhs) as one strategy to suppress antiviral responses [1]. At the same time, sufficient protein synthetic capacity must be retained to allow viral replication. This balance is often achieved through a combination of factors, inhibiting or promoting major steps of protein synthesis [2]. Therefore, despite a general inhibition of host protein synthesis, some host proteins bypass this global inhibition and are selectively expressed throughout the course of the infection [3]. 

The main aims of this study are the proof of the applicability of the dynamic SILAC approach to measure the vhs in general, and the characterization of the extent and the specificity of ASFV-induced vhs in the natural primary target cell. The measurement of protein synthesis is a prerequisite for the detailed characterization of vhs. In the past, the large-scale determination of protein synthesis rates was usually achieved based on metabolic radioactive labeling and a combination of two-dimensional gel electrophoresis with MALDI-TOF mass spectrometry (MS). Modern highly sensitive quantitative MS-based approaches such as the incorporation of ‘clickable’ biorthogonal amino acids into the growing peptide chain [4] or isotope labeling with amino acids in cell culture (SILAC) [5] allow one to analyze protein synthesis without the need for radioactive reagents. SILAC is based on the metabolic incorporation of heavy isotopomers of natural amino acids into the newly synthesized protein during cell culture. In the dynamic (or pulsed) SILAC approaches, isotope labels are added to the cell cultures in the course of the experiment [6,7]. The determination of isotope ratios then allows the calculation of protein synthesis or degradation rates (for a recent review see [8]). Dynamic SILAC has been applied to investigate protein turnover rates in different cell types [9], to assess the effects of chemical inhibitors and cell differentiation on protein synthesis [10], and to measure the protein turnover of Salmonella [11]. In the context of viral infections, dynamic SILAC approaches have been applied to analyze synthesis rates of host proteins after human immunodeficiency virus (HIV) infection [12], to analyze influenza A virus protein synthesis [13], and to investigate HCMV-induced protein degradation [14]. However, to our knowledge, this approach has not been used to quantify either the extent or the specificity of a virus-induced shutoff.

Vhs has been studied extensively for RNA viruses, such as influenza virus, or DNA viruses, such as the herpesviruses or VV. Even though these viruses differ in genome organization, replication mechanisms, compartments, targeted cell types, and host species, they have evolved similar molecular mechanisms targeting the host protein synthesis machinery (reviewed in [15,16]). These viral strategies include, among others, the interference with mRNA maturation (splicing and polyadenylation) and nuclear export, the reduction of cellular mRNA levels through decapping-mediated degradation, and the regulation of cap-dependent translation initiation (reviewed by [2]) [17,18,19,20].

The alphaherpesviruses, with their short lytic replication cycles, have been extensively studied to reveal the mechanisms of vhs, and very detailed information is available for the human herpesvirus-1 (HHV-1, or herpes simplex virus-1, HSV-1) [15]. Suid herpesvirus-1 (Pseudorabies virus, PrV), an alphaherpesvirus, such as HSV-1, is the causative agent of Aujeszky’s disease [21] in pigs, but also infects a wide range of other mammals [22]. We have included PrV in this study as it is an important swine pathogen that can infect porcine macrophages [23,24], such as ASFV, and represents a virus with a well documented vhs [25,26,27]. Several alphaherpesvirus proteins have been identified as modulators of protein expression favoring or counteracting the vhs [28]. A common mechanism to counteract vhs is the maintenance of eIF2α in a dephosphorylated state [2]. In the herpesviruses, US11, gB, and ICP34.5 or IE180 (in the case of PrV) contribute to this mechanism [28]. 

African swine fever is a deadly disease affecting members of the *Suidae* family. It is caused by African swine fever virus (ASFV, family *Asfarviridae*). Currently, neither a vaccine, nor a treatment, is available for the disease, which has been repeatedly introduced into Europe and America in the past decades [29]. The most recent introduction to Georgia in 2007 resulted in a panzootic spread of the virus [30].

Similar to PrV, ASFV is a large virus containing a dsDNA genome. ASFV encodes more than 150 genes, and, among them are structural proteins, which are proteins involved in virion morphogenesis and evasion of host defense mechanisms. However, many of the gene products lack detailed functional characterization, and functions are often only predicted based on homology comparisons to other viruses, if available (reviewed in [31]). The multi-faceted influences of ASFV infection on cell homeostasis have been summarized in a recent review [32]. ASFV infection interferes with the host cell transcription and modulates the expression and activity of specific target genes [33,34,35]. While some studies report a general vhs affecting up to 65% of cellular proteins [34,36], others did not observe any shutoff [37,38,39]. In a screen for ASFV proteins affecting the global protein expression [40], a wide range of effects was observed after expressing single viral proteins in HEK-293T cells from slight enhancement to a strong suppression with the maximum of 15-fold suppression exerted by pE66L. As the mechanism for the pE66L-induced vhs, the suppression of translation through the PKR/eIF2α pathway was identified. In this study, the expression of a marker protein or a ribopuromycylation assay was used to measure the global protein expression and protein biosynthetic activity, respectively. Notably, the expression of 14 viral proteins resulted in over five-fold suppression of marker expression, indicating that multiple mechanisms of ASFV-mediated vhs may exist. A function of pDP71L, a homolog of herpes-viral translation enhancer ICP34.5 [41], in the dephosphorylation of eIF2α and the maintenance of translation, has been shown [42,43].

Studies of the cellular responses to ASFV infection identified several ASFV genes potentially involved in the modulation of cellular transcription. These include the IkB-like protein pA238L, the translation enhancer pDP71L, and the mRNA modifying enzymes pNP868R, pI267L, pD250R, pEP424R, and pC475L [17,18,44,45,46]. Additionally, an influence of ASFV infection on subnuclear domains and chromatin architecture has been observed, potentially suppressing host gene expression by affecting transcription through altered methylation patterns of histones [47]. However, it is assumed that more viral proteins can potentially influence the cellular protein synthesis, and ASFV infection may affect hitherto unknown cellular pathways [31]. 

While previous studies investigating ASFV-induced effects on host protein synthesis used radioactive labeling, electrophoretic methods, and chemical controls [33,34], we applied dynamic SILAC labeling in combination with MS to evaluate the proteome-wide effect of ASFV on the synthesis rate of host proteins in primary porcine monocyte-derived macrophages. Dynamic SILAC approaches have been applied to characterize the biological half-lives of proteins in different cell types and to estimate protein synthesis rates in response to various stimuli. To our knowledge to date, a dynamic SILAC approach has not been used to quantify the strength or the specificity of virus-induced host cell shutoff. 

## 2. Materials and Methods

### 2.1. Cell Lines and Viruses

Cell lines were obtained from the Biobank of the Friedrich-Loeffler Institut (FLI). The porcine cell line SPEV (domestic pig kidney cell line; CCLV-RIE #0008) cultured in Eagle’s minimum essential medium with Hanks’ salts (MEM-H) supplemented with 10% FCS was used for stock growth of PrV and PrV infection kinetics. 

The shutoff-competent PrV strain Kaplan mutant PrV-ΔgG-GFP (hereafter referred to as PrV) has been described previously [48]. PrV titers were determined on Madin-Darby Bovine Kidney (MDBK) cells (CCLV-RIE #0261) cultured in MEM-H supplemented with 10% FCS.

Wild-type ASFV-isolate “Armenia 2008” [49] (hereafter referred to as ASFV) was passed three times on peripheral blood monocytic cells (PBMC) cultured in Iscove′s modified Dulbecco’s medium mixed with Ham’s F-12 nutrient mix (1:1; *v*/*v*) supplemented with 10% FCS and 1% Penicillin/Streptomycin solution (ThermoFisher Scientific, Dreieich, Germany). ASFV titers were determined on PBMCs. Infections with ASFV were carried out in a biocontainment facility that fulfills the safety requirements for ASF laboratories and animal facilities.

### 2.2. Isolation and Cultivation of Monocyte-Derived Macrophages

Blood was drawn from 9- to 12-month-old domestic pigs kept at the FLI animal facility (LALLF-Nr. 7221.3-2-041/17). The isolation and cultivation of monocyte-derived macrophages for MS analysis has recently been described [50]. Briefly, PBMCs, obtained using Pancoll animal density gradient medium (density 1.077 g/mL, PAN-Biotech, Aidenbach, German), were selected for CD172a+ cells using BD anti-Mouse IgG1 magnetic particles (BD Biosciences, Heidelberg, Germany) in combination with α-SWC3 hybridoma supernatant (clone 74-22-15, provided by U. Blohm, FLI, Greifswald, Germany) according to the recommendations of the manufacturers. CD172a+ PBMCs were cultured in Primaria^TM^ Cell Culture dishes (Corning, Wiesbaden, Germany) at 37 °C, 2.5% CO_2_. To induce differentiation, fresh culture medium supplemented with 5 ng/mL porcine GM-CSF (KingFisher Biotech, Saint Paul, MN, USA) was added one day after isolation.

### 2.3. PrV Growth Kinetics

For PrV growth kinetics, monocyte-derived macrophages (moMΦ) (1 × 10^6^ cells/well) cultured in Iscove′s modified Dulbecco’s medium mixed with Ham’s F-12 nutrient mix (in equal parts), supplemented with 10% FCS and 1% Penicillin/Streptomycin solution and SPEV cells (2.5 × 10^5^ cells/well), were infected with PrV at MOI 0.1. Cells were inoculated for 1 h on ice (SPEV) or 30 min at 37 °C (moMΦ). The inoculum was then replaced by fresh culture medium, and cells were incubated at 37 °C, 2.5% CO_2_. After 2, 6, 12, 24, and 48 h, viral titers and PrV protein expression were determined by titration on MDBK cells and PrV-gB-specific immunoblots.

Cells were lysed in 50 µL lysis buffer (2% SDS in 0.1 M Tris-HCl, pH 8.0) after washing with PBS trice. To enhance the lysis and inactivation of viruses, samples were incubated at 95 °C for 10 min, cooled to RT, and clarified by centrifugation (5 min, 10,000× *g*, 20 °C). The resulting supernatant is referred to as “lysate”.

### 2.4. SDS PAGE, Immunoblotting and Immunofluorescence Analysis 

Cell lysates were separated by gradient (7–15%) acrylamide gel electrophoresis [51], followed by Coomassie Brilliant Blue staining [52] or immunoblotting [53]. Viral proteins were detected using rabbit sera specific for PrV-gB or ASFV-p30 and ASFV-p72 (provided by W. Fuchs, FLI) and visualized by chemiluminescence after incubation with a peroxidase-conjugated anti-rabbit antibody and Clarity Western ECL substrate (BioRad, Feldkirchen, Germany).

### 2.5. Generation of SILAC Samples for MS Analysis

For the generation of MS samples, moMΦ were cultured in SILAC RPMI medium (Silantes, München, Germany) supplemented with 10% dialyzed FCS, 0.855 mol/L L-leucine, and 1.15 mol/L L-arginine. SILAC medium supplemented with light isotopes (^12^C_6_ lysine, ^12^C_6_ arginine) was used after isolation and during differentiation. For the inoculation with ASFV (MOI 1) or PrV (MOI 2) and cultivation during infection, SILAC RPMI medium supplemented with ^13^C_6_ lysine (>98% isotope purity, Silantes) and ^13^C_6_ arginine (>98% purity, Silantes) (heavy SILAC-medium) was used. 

To enhance infection rates during ASFV infection, moMΦ were centrifuged during inoculation (60 min, 37 °C, 600× *g*) as described [50]. Inoculation of moMΦ with PrV was carried out for 30 min at 37 °C. The inoculum was then removed, and cells were washed three times with PBS before the addition of fresh heavy SILAC medium.

PrV-infected moMΦ were lysed 16 hpi as described above. Mock- and ASFV-infected moMΦ were harvested at 24 hpi. ASFV-infected moMΦ were detached using cold 50 mM EDTA in PBS, pelleted (250× *g*, 5 min, 4 °C), and lysed in lysis buffer. For each sample group, three biological replicates were collected.

### 2.6. MS-Analysis of SILAC Samples

Reduced cell lysates (final concentration DTT 0.5%, incubation: 10 min, 95 °C) of SILAC samples were digested by Filter Aided Sample Preparation (FASP) as described [54] at a trypsin-to-substrate ratio of 1:50. Desalted peptides were solubilized in 0.1% formic acid (FA). Per sample, 500 ng of peptides were analyzed using a nanoElute^®^ HPLC coupled to a TimsTOF Pro instrument (both Bruker, Bremen, Germany). Peptides were separated at 40 °C and 400 nL/min flow rate on an IonOpticks Aurora column (25 cm × 75 µm ID, 1.6 µm C18) over a 115 min gradient (2–15% solvent B (0–60 min), 15–24% solvent B (60–90 min), 24–34% solvent B (90–105 min), 35–95% solvent B (105–107 min), and 95% solvent B (107–115 min). Solvent A was 0.1% FA, and solvent B was 0.1% FA in acetonitrile. The TimsTOF Pro instrument was equipped with a CaptiveSpray ion source (Bruker) and operated in Parallel Accumulation and Serial Fragmentation (PASEF) mode using the standard method for proteome analysis (1.1 s cycle time), recommended by the manufacturer.

Database queries for protein identifications were conducted with the Fragpipe [55] search engine using a sequence database compiled from the host (*S. scrofa*; downloaded from Ensembl repository [56]) and the proteomes of ASFV-Georgia, (GenBank FR682468.2) or PrV-Kaplan (Genbank NC_006151) [57], respectively. The false discovery rate (FDR) was set to 0.1%, 2 missed cleavages were tolerated, carbamidomethylation of cysteine residues was set as fixed modification, and oxidation of methionine, protein N-terminal amidation, and phosphorylation of serine, threonine, and tyrosine were chosen as optional modifications. 

The MS data have been deposited to the ProteomeXchange Consortium (http://proteomecentral.proteomexchange.org accessed on 2 February 2023) via the PRIDE partner repository [58] with the dataset identifier PXD039806.

### 2.7. Data Analysis

Identification and quantification results from Fragpipe were processed in R [59] using the package data.table [60]. Porcine identifiers were referenced to HGNC (HUGO Gene Nomenclature Committee) symbols [61] with gProfiler [62]. Relative synthesis rates (Syn_rel_) were defined as the ratio of newly synthesized protein over the pre-existing amount of protein and calculated based on the experimental heavy-to-light ratios (HoL) using the formula: Syn_rel_ = HoL/(1 − q × HoL). Here, q is a correction factor estimating the effective isotope purity to account for the isotope purity of the reagents and any residual unlabeled amino acids remaining in the cell at the time of medium replacement. Evaluation of the top five ASFV and PrV proteins with the highest number of detected peptides determined q as 0.0873.

Statistical analysis was performed with R (version 4.1.1) and Perseus software v1.6.15.0 [63]. The R package ggplot2 [64] was used for the construction of graphics, and the enrichment analysis of Gene Ontology (GO) terms [65] and Kyoto Encyclopedia of Genes and Genomes (KEGG) pathways [66,67] was performed in R using the gProfiler package. 

## 3. Results

**Figure 1:**
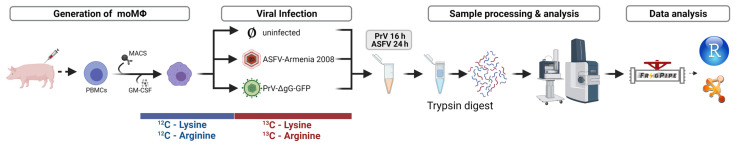
To assess ASFV-induced shutoff, moMΦ were isolated and differentiated, as described [50]. Simultaneously to infection with ASFV or PrV, the medium containing (conventional) ^12^C_6_-Lysine and ^12^C_6_-Arginine was replaced by a medium containing ^13^C_6_-Lysine and ^13^C_6_-Arginine instead. PrV-infected samples were incubated for 16 h, while mock- and ASFV-infected samples were harvested after 24 h. Whole-cell lysates were processed using a standard bottom-up proteomic workflow, and MS data were analyzed using Fragpipe, in-house scripts in R, and Cytoscape (version 3.9.1) software.

To generate SILAC samples, moMΦ were inoculated with ASFV, PrV, or cell culture medium and cultivated in medium containing ^13^C_6_-labeled lysine and arginine. At late stages of infection, whole cell lysates were collected, digested to tryptic peptides, and analyzed using a TimsTof Pro MS platform (Figure 1).

Of the 3831 porcine gene products that were identified in total, relative synthesis rates could be calculated for 2813, 2728 in mock-infected cells, 2216 in ASFV-infected cells, and 2685 in PrV-infected cells (Table S1). 

**Figure 2:**
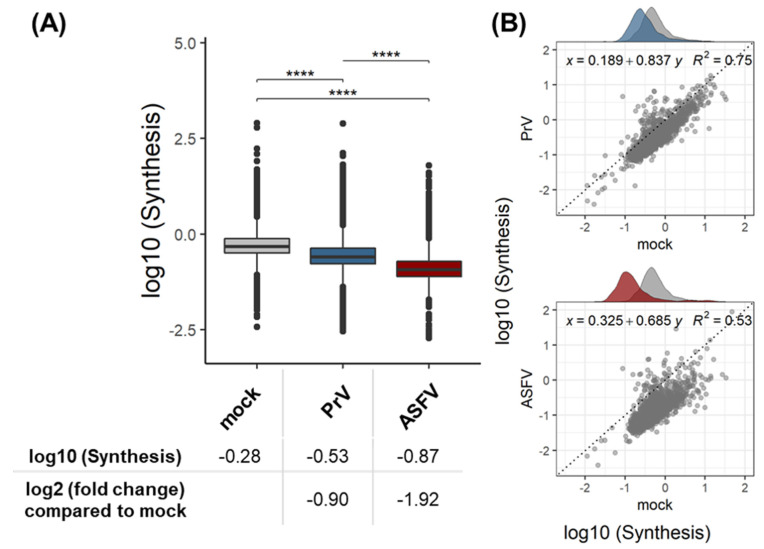
(**A**) Log10 of relative synthesis rates of host proteins in mock-, PrV-, and ASFV-infected moMΦ. (**B**) Comparison of relative synthesis rates of host proteins in ASFV- or PrV-infected moMΦ compared to mock-infected moMΦ. The dissection line is indicated as a reference. Data represent means of three biological replicates per group. ****: *p* ≤ 0.0001. Grey—mock-infected moMΦ; red—ASFV-infected moMΦ; blue—PrV-infected moMΦ.

For the viral proteins, the expected high heavy-to-light ratios and consequently high relative synthesis rates were measured, and the means of the log10-transformed relative synthesis rates were set to protein synthesis rates of 100% (proteins not being present before infection and entirely synthesized beginning with the infection). In contrast, relative synthesis rates of host proteins were lower, and this was also true in mock-infected moMΦ (mean log10(relSyn) = −0.28) (Figure 2A).

### 3.1. Validation of the Dynamic SILAC Approach for the Evaluation of Vhs Using PrV

As macrophages are not the primary target cells of PrV, the expression of the PrV glycoprotein B (gB) and viral titers were monitored over 48 hpi to confirm the ability of PrV to productively infect the selected cell populations (Figure S1A,B). PrV infection was verified by immunoblot against PrV-gB (Figure S1C) and by mass-spectrometric detection of 31 PrV proteins (Supplementary Table S1) in the cell lysates. In PrV-infected moMΦ, an approximately two-fold mean reduction in protein synthesis rates compared to mock-infected moMΦ was observed (Figure 2A). In the correlation analysis (Figure 2B), this reduction manifested as an apparently linear shift of the distribution away from the dissecting line and a slope of the correlation coefficient smaller than 1.

These results obtained after infection with PrV, a well described vhs inducer, showed that the dynamic SILAC approach is suitable to quantitatively evaluate a vhs.

### 3.2. Quantitative Evaluation of the ASFV-Induced Vhs

Following the validation of the experimental setup, the ASFV-induced vhs was evaluated. As for PrV, infections with ASFV were verified by immunoblot (Figure S1C) and by mass-spectrometric detection of 122 ASFV proteins (Supplementary Table S1) in the cell lysates. 

The correlation analysis of mock- and ASFV-infected moMΦ indicated the presence of a strong vhs, due to the low correlation coefficient (R^2^ = 0.53) and low slope (slope = 0.685) (Figure 2B). Statistical analysis confirmed this first impression and revealed an on average almost four-fold reduction of host protein synthesis rates compared to mock-infected moMΦ (Figure 2A).

**Figure 3:**
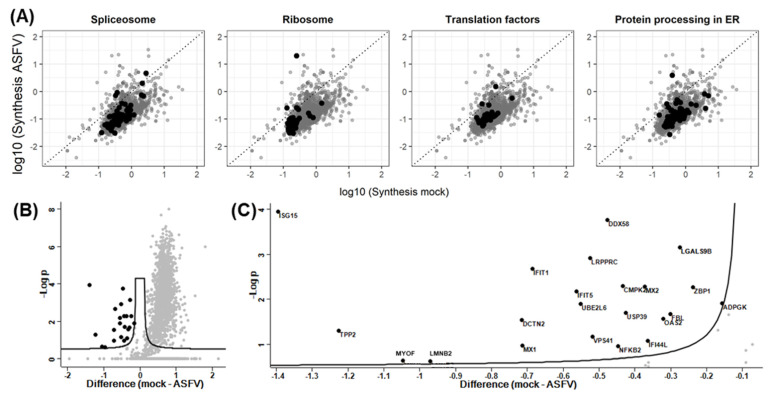
(**A**) Correlation plot comparing the mean (n = 3) protein synthesis rates observed in mock- and ASFV-infected moMΦ. Genes associated with the spliceosome, ribosome, translation factors, and protein processing in the ER, respectively, are highlighted in black in the four panels. (**B**) Volcano plot representation of statistical analysis performed in Perseus software, highlighting genes with significantly increased synthesis rates after infection. An amount of 22 genes were upregulated, and 1621 were downregulated. The *x*-axis is log2-scaled, and the *y*-axis shows the negative log10 of the *p*-values. (**C**) Magnification of panel B, including labeling of genes with increased synthesis rates.

Initially, no outstanding effects on the synthesis rates or expression levels of genes involved in mRNA processing (splicing), translation (ribosome and translation factors), or protein processing were observed (Figure 3A). 

While the synthesis rate of 74.7% of genes (1621 genes of 2170 quantified genes) was significantly reduced following ASFV infection, only 22 proteins had significantly upregulated synthesis rates (Figure 3B). These included several genes involved in the IFN-response, such as MX1, MX2, ISG15, DDX58, and ZBP1, as well as UBE2L6, USP39, OAS2, IFIT1, and IFI5 (Figure 3C).

Additionally, 676 genes were not synthesized in ASFV-infected moMΦ, and 47 genes were exclusively synthesized in ASFV-infected moMΦ (Table S1).

**Figure 4:**
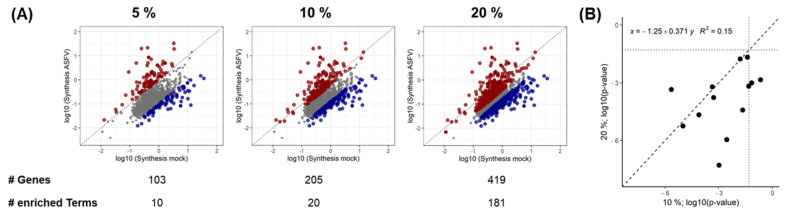
Term enrichment analysis. (**A**) Visualization of the percentiles used for the analysis. The dissecting line is dotted. Red—increased synthesis; blue—decreased synthesis. (**B**) Correlation of *p*-values of terms enriched using the 10 and 20% percentile. The intersecting line is dashed, and the dotted lines indicate *p*-values of 0.05.

To obtain a more detailed view of pathways impacted by the shutoff or by infection-induced stimulation of protein synthesis, KEGG and Reactome pathway enrichment analyses was performed, based on the genes showing the strongest shutoff or stimulation. To estimate the sensitivity of the results to the choice of a cutoff value, the 5%, 10%, and 20% percentiles were tested as cutoffs (Figure 4). As expected from the distribution of synthesis rate values in Figure 4A, the most stringent cutoff (5%) was very sensitive to detect enriched terms in the population with increased synthesis rates only. For further evaluation, the 10% percentiles were chosen as the inclusion of even more genes with the 20% percentile cutoff yielded similar results, although with slightly different *p*-values (Figure 4B and Table 1).

For the evaluation of enriched terms with gProfiler, the qualitative data were added to the gene lists calculated based on the 10% percentiles of synthesis rates in order to include genes represented by proteins that either had dropped below detection limits after infection (676 genes) or were exclusively detected after ASFV infection (47 genes). The underlying gene lists and the complete results of the enrichment analysis are available in Tables S2 and S3. A summary of the gProfiler results is given in Table 2.

The enriched terms relating to the immune system, membrane trafficking, RNA metabolism, and functions of the nucleus are shown. Remarkably, terms representing antiviral mechanisms mediated by interferon and cytokine signaling were enriched in the query of proteins with the highest synthesis rates. These mechanisms also included ISG15-mediated antiviral responses.

In contrast, the query based on proteins, which were subject to the most efficient shutdown and thus showed the strongest decrease in synthesis rates produced enrichment of pathways related to the RNA metabolism (especially the splicing process), the nucleocytoplasmic transport and other functions of the nucleus, and the regulation of necroptotic cell death. 

### 3.3. Effect of Synthesis Rates on Protein Abundance

The chosen experimental approach allowed not only the quantitative analysis of synthesis rates, but also the evaluation of absolute protein abundances based on label-free quantification. Setting the fold changes of synthesis rates with the fold changes of expression levels enables the evaluation of the effects of synthesis rates on expression levels.

**Figure 5:**
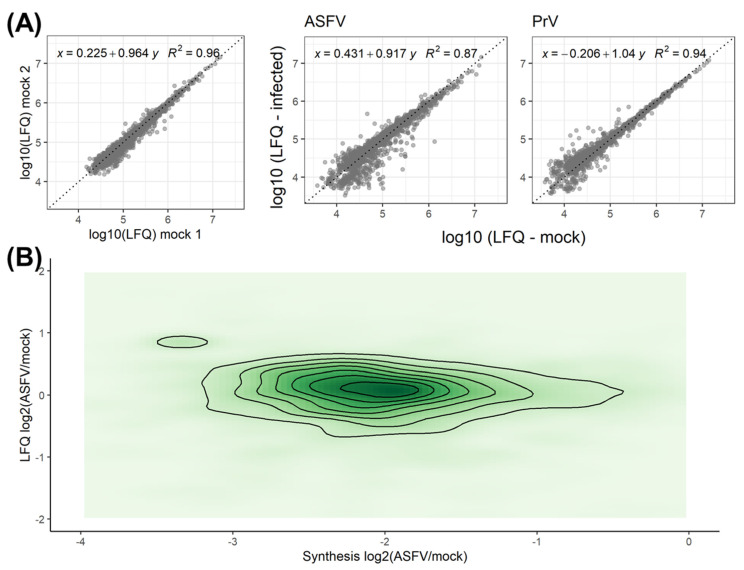
(**A**) Correlation analysis of absolute expression levels of host proteins based on label-free quantification (LFQ) between mock-infected moMΦ (left) and ASFV (middle) or PrV-infected (right) moMΦ compared to mock-infected moMΦ. (**B**) Contour plot showing the relation of synthesis rates (synthesis, *x*-axis) and fold changes of absolute protein abundances based on label-free quantification (LFQ, *y*-axis) of porcine proteins expressed in ASFV-infected moMΦ compared to mock-infected moMΦ (n = 3).

High correlation coefficients between the expression levels of proteins before and after infection (0.87 and 0.94 for ASFV and PrV infection, respectively) indicated that no major changes in expression levels were induced by either virus, even though some variability among genes with low expression levels was noted in ASFV-infected moMΦ (Figure 5A). 

When setting the change in the absolute expression levels in relation to the alteration of synthesis levels, it stands out that a wider range of alterations can be noted when evaluating synthesis rates compared to absolute protein levels (Figure 5B).

**Figure 6:**
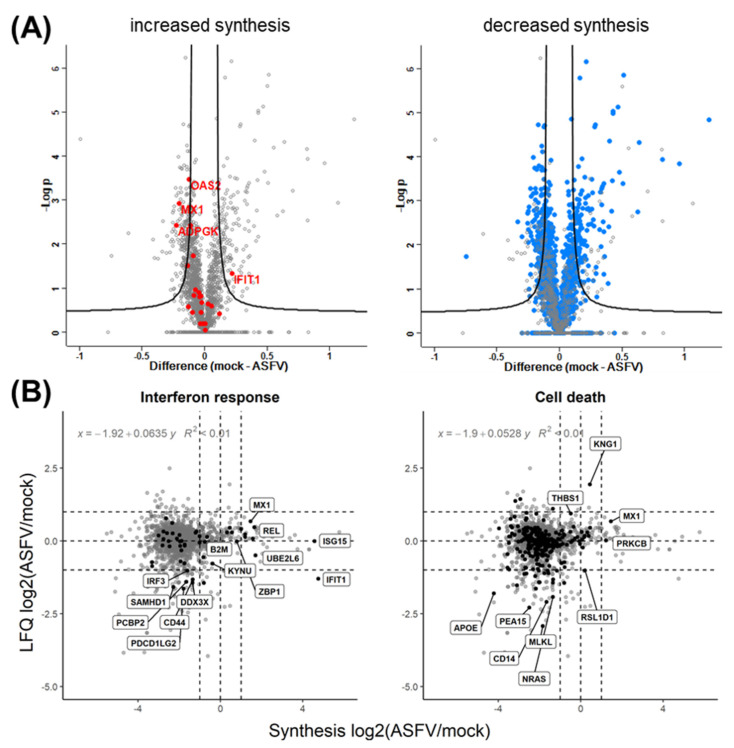
(**A**) Analysis of protein levels after LFQ quantification. Volcano plots show fold changes in protein abundances on the x-axes and −log10(*p*-values) on the y-axes. Genes with significantly increased (red) or decreased (blue) synthesis rates are highlighted. (**B**) Correlation of relative synthesis rates and protein abundance levels in mock-infected and ASFV-infected moMΦ. Relative protein synthesis rates (ASFV-infected/mock-infected, based on the SILAC measurement) and relative protein abundance levels (ASFV-infected/mock-infected, based on LFQ measurement) are presented as log2 values. Dotted horizontal and vertical lines indicate zero change and two-fold changes in either direction. In the left and right panels, genes related to the interferon response and cell death are highlighted, respectively. Note that (i) the range of synthesis rates spans approximately 8 logs, while the span of abundance levels is roughly 4 logs, and (ii) the distribution of relative synthesis rates centers around approximately −2 (also see Figure 1), whereas the relative abundances center around 0. There is no striking correlation between changes in relative synthesis rates and relative protein abundance.

This impression was confirmed by the statistical analysis with the majority of cellular proteins showing no statistically significant changes in abundance. The observed changes in expression levels appear to be independent of changes in the synthesis rates for many proteins, as only 124 of the 1603 proteins with reduced synthesis also had reduced expression levels (Figure 6A).

Of the genes with significantly increased synthesis rates (Figure 3C), only four had significantly altered expression levels. While increased synthesis rates of OAS2, MX1, and ADPGK were accompanied by increased expression levels, IFIT1, despite the increased synthesis, showed reduced expression levels (Figure 6A, left panel). For other genes involved in the interferon response with increased synthesis rates, no effects on the expression levels could be noted, among them ISG15 (Figure 6B). In contrast to the situation after infection with PrV, increased synthesis rates of proteins involved in the interferon response did not result in the expected augmented protein levels after ASFV infection (Figure 6B and Figure S2).

## 4. Discussion

Inhibition of host protein synthesis by viral infections has been described and characterized for many viruses, such as vaccinia-, corona-, influenza-, and herpesviruses [15,16,68,69]. The wide distribution among very diverse viruses indicates that viruses strongly benefit from the redirection of the cellular machinery for the expression of their own genes and the suppression of the antiviral response, which is effectuated by vhs. Even though the phylogenetic distance between certain viruses may be large, the mechanisms to target host protein synthesis can be similar [15]. However, vhs does not affect every gene to the same extent but the inhibition of expression rather seems to be selective [3]. Therefore, open-view approaches that are capable to measure gene expression on a large scale, such as mRNA-sequencing and MS, are required to explore the correlation of vhs with cellular functions and thus increase our understanding of the mechanisms that viruses implement to undermine their host cells’ capacity to express genes.

Proteomic studies analyzing the host protein expression after ASFV infection on a global scale are very limited [33,37,50,70,71,72,73], and systematic studies of the specificity of ASFV-induced vhs using high throughput approaches are lack to date. In the early two-dimensional gel electrophoretic studies, protein identification was either impossible or limited due to the MS instrumentation available at the time. Thus, an ASFV-induced shutoff could be shown by combination of two-dimensional gel electrophoresis with metabolic radioactive protein labeling, but either the proteins of interest were not identified [37,73] or the number of identified proteins was low [33]. Additionally, Vero cells infected with ASFV-BA71V were used in these studies so that data from the natural host or the primary target cells of ASFV infection, macrophages, are not available. For this study, we have prepared monocyte-derived macrophages from blood, which can be obtained without euthanizing a pig. Besides the need to differentiate these cells before they can be used for ASFV infection, another major drawback of this method (in contrast to preparations from lung lavages) are low yields [74] of these non-dividing cells. However, due to the high sensitivity of the MS, this was acceptable. Additionally, the higher experimental variability of gene expression patterns that could be expected in moMΦ compared to cultured stable cell lines [50] was accepted for the benefit of data originating from the infection of the primary target cell of ASFV in the pig.

To provide the first systematic characterization of the ASFV-induced vhs, we have applied the dynamic SILAC workflow, a quantitative MS-based approach relying on the incorporation of heavy isotope-labeled amino acids into proteins to analyze the protein synthesis in ASFV-infected primary moMΦ. This setting allowed the large-scale analysis of ASFV-induced shutoff in the natural target cells avoiding hazards and precautions coming with the use of radioactive labeling or the use of unphysiological reagents, such as clickable biorthogonal amino acid homologs. At the same time, the effect of the infection on the synthesis rates can be quantified for single host proteins, allowing us to identify individual proteins with synthesis rates that are downregulated, remain unchanged, or are induced by ASFV infection. Such basic knowledge may serve to understand pathogenicity and to develop antiviral strategies. The importance of pE66L in the PKR/eIF2α-mediated shutoff has been described by Shen et al. [40]. In this publication, it was also demonstrated that titers of an E66L knockout mutant did not significantly differ from those of the corresponding ASFV wild-type. Although, in the absence of pE66L, a partial recovery of the host cell synthesis and increased RNA levels of several immune-related genes, such as interferon beta 1, were observed. Thus, the importance of vhs for the replication of ASFV remains partially unclear. 

As positive controls, moMΦ were infected in parallel with Suid herpesvirus-1 (SuHV-1, PrV), a swine pathogen with a well documented vhs. The presented results confirm an ASFV-induced vhs, which, on average, is stronger than observed after PrV infection. The applied dynamic SILAC is a straightforward and flexible approach to quantify vhs that can be applied to a variety of in vitro infection models. The major possible restriction is the availability of a cell culture medium that supports the culture and infection of the studied cells, which is free of the conventional amino acids used for labeling. This could be a hurdle for cells requiring very demanding cell culture conditions. Usually, a tryptic digest will be performed for the proteolysis, and the labeled amino acids will consequentially be Lysine and Arginine, two amino acids that are readily available as ^13^C isotopomers.

Different mechanisms have been proposed to explain the impact of ASFV infection on the expression of host proteins. Castello and colleagues [34] hypothesized that the observed ASFV vhs could be attributed to the redistribution of the translation machinery and the degradation of polyadenylated mRNAs, which vanish from the cytoplasm of infected cells at the late stage of infection [34]. Alternatively, the increased expression levels of ASFV genes could result in decreased cellular gene expression [75].

We observed that the synthesis of proteins involved in mRNA splicing and nuclear export of mature transcripts was decreased (Table 2). This might indicate that the maturation of transcripts is modulated, and transcripts might be retained within the nucleus. Alternatively, cellular mRNAs could be degraded, as has been suggested as a mechanism for the herpesviral vhs protein pUL41 [25,76], or made inaccessible for the translation machinery, as was described for the coronavirus NS1 protein, which blocks the ribosome to selectively favor the translation of viral RNAs [77,78]. Currently, no ASFV protein having such functions has been identified. However, ASFV D250R is known to encode a decapping enzyme that exposes mRNAs to rapid degradation [17,79].

Modulation of transcript maturation and modification is a common feature of viral infections for example mediated by herpesviruses, influenza viruses, and VV [15,16]. While the overall RNA expression seems to be unaffected by ASFV infection [39,80], specific effects on the expression levels of limited numbers of proteins have been noted [34,50,81]. However, as we show in Figure 5B and Figure 6B, altered synthesis rates did not generally correlate with altered expression levels, which is a known phenomenon [82]. As the range of changes observed for the synthesis rates was much wider than for the protein levels (Figure 5B), we conclude that the measurement of expression levels is not a sensitive approach to quantify vhs. Moreover, our data show that very stable expression levels are maintained after ASFV and PrV infection, suggesting that not only protein synthesis, but also protein degradation, may be targeted by infection with either virus (Figure 5B). The extensive retardation of protein turnover in the context of a virus infection may have general effects on the proteome, which are related to protein aging, such as misfolding, the introduction of post-translational modifications (oxidation or deamidation), and, finally, aggregate formation. In this context, recent findings connecting protein age with protein stability [83], and showing biphasic degradation of cellular proteins with a rapid initial phase and a slower later phase of degradation, should be mentioned. If this concept holds also for virus-infected cells, a virus-induced shutoff could automatically lead to an apparently higher stability of the proteome caused by the lack of newly synthesized proteins that are subject to rapid degradation.

Interestingly, most genes with significantly increased synthesis rates, among them ISG15, OAS1, DDX58, UBE2L2, MX1, MX2, IFIT1, and IFI5, are strongly expressed in response to interferons as a consequence of dsRNA sensing (Figure 3C) [84]. The elevated synthesis levels of these interferon-stimulated genes are likely accompanied by an ASFV-induced increase in mRNA levels [75,85,86], even though none of them, except MX1, showed significantly increased expression levels (Figure 6B). However, these observations correlate with previous studies made on transcript and protein levels for MX1 and ISG15 [72,80].

In this context, the inability of ASFV to control MX1 expression levels is remarkable as inhibitory effects of MX1 expression on ASFV morphogenesis have been described [87]. It would be interesting to investigate if and how ASFV interacts with and modulates MX1 activity to prevent adverse effects on viral replication.

This points out two important characteristics of ASFV infections. First, the expression of selected genes of the immune response is strongly induced after ASFV infection, despite the ASFV-mediated inhibition of signaling pathways, such as cGAS-STING, IRFs, and NFkB, which promote their expression [88,89,90], while the expression of other genes is strongly decreased (Table 2). Secondly, the lack of effect on total protein expression levels despite the decreased synthesis rates indicates the presence of ASFV-encoded mechanisms to stabilize proteins, for instance, by targeting cellular ubiquitin-mediated degradation. ASFV proteins possibly involved in these processes include serine-threonine kinase pR298L [91,92,93] and the ubiquitin-conjugating enzyme pI215L [94]. pI215L may play a central role in the modulation of cellular protein expression, as it has also been described to be involved in the maintenance of mTORC1 activity and the availability of eIF4G for translation initiation [94,95].

Other cellular pathways potentially influenced by an ASFV infection to modulate protein stability include ubiquitin-like modifications, such as ISGylation and SUMOylation. Indeed, several genes affected by or involved in ISGylation, such as MX1, DDX58 (RIGI), and UBE2L2 [84,96] did stand out due to their significantly increased synthesis (Figure 3C). 

Overall, the MS-based analysis of ASFV-infected moMΦ using a dynamic SILAC approach provided detailed insight into the control of cellular protein synthesis and protein levels by ASFV. The observed massive and selective changes in synthesis rates with concomitant stability of most protein levels suggest that this control is very complex and requires high-throughput techniques for their elucidation. The wide dynamic range we have observed for dynamic SILAC measurements allows the detection of pathways involved in ASFV infection with high sensitivity. Thus, this approach seems to be an excellent tool to unravel the contributions of different ASFV proteins, or possibly other factors, such as host species, cell type, or ASFV genotype, in relation to the control of host gene expression by ASFV. 

## Figures and Tables

**Table 1:** Comparison of enriched KEGG and Reactome (REAC) terms using the 5%, 10%, and 20% percentiles, corresponding to 103, 205, and 419 genes, respectively. Terms that are not enriched under the respective conditions, but in the complementary gene subsets (increased/decreased synthesis) of the same percentile, are marked as ‘1’, and ‘-’ indicates terms that are not enriched in the analyzed percentile.

Term ID	Term Name	Increased Synthesis			Decreased Synthesis		
		5%	10%	20%	5%	10%	20%
KEGG:00010	Glycolysis/Gluconeogenesis	-	3.20 × 10^−2^	-	-	1	-
KEGG:00511	Other glycan degradation	-	1	1	-	2.88 × 10^−3^	1.12 × 10^−6^
KEGG:00970	Aminoacyl-tRNA biosynthesis	-	2.95 × 10^−2^	-	-	1	-
KEGG:01200	Carbon metabolism	-	2.25 × 10^−2^	-	-	1	-
KEGG:04142	Lysosome	-	1	1	-	6.92 × 10^−6^	3.34 × 10^−17^
KEGG:04612	Antigen processing and presentation	-	4.11 × 10^−2^	2.11 × 10^−2^	-	1	1
KEGG:05162	Measles	2.99 × 10^−2^	1.55 × 10^−2^	-	1	1	-
KEGG:05164	Influenza A	1.16 × 10^−2^	1.34 × 10^−2^	-	1	1	-
KEGG:05169	Epstein-Barr virus infection	3.08 × 10^−2^	4.82 × 10^−2^	6.68 × 10^−4^	1	1	1
KEGG:05171	Coronavirus disease—COVID-19	7.85 × 10^−3^	2.22 × 10^−2^	3.84 × 10^−5^	1	1	1
REAC:R-HSA-1169408	ISG15 antiviral mechanism	2.44 × 10^−4^	4.44 × 10^−4^	6.19 × 10^−4^	1	1	1
REAC:R-HSA-1169410	Antiviral mechanism by IFN-stimulated genes	2.98 × 10^−5^	1.01 × 10^−5^	5.69 × 10^−6^	1	1	1
REAC:R-HSA-1280215	Cytokine Signaling in Immune system	2.96 × 10^−3^	1.05 × 10^−3^	5.27 × 10^−8^	1	1	1
REAC:R-HSA-168249	Innate Immune System	-	4.30 × 10^−1^	2.85 × 10^−3^	-	1.30 × 10^−2^	1.57 × 10^−8^
REAC:R-HSA-168256	Immune System	3.69 × 10^−2^	7.77 × 10^−5^	3.27 × 10^−7^	1	1	4.30 × 10^−5^
REAC:R-HSA-379716	Cytosolic tRNA aminoacylation	-	5.21 × 10^−4^	1.72 × 10^−4^	-	1	1
REAC:R-HSA-379724	tRNA Aminoacylation	-	1.62 × 10^−2^	1.70 × 10^−2^	-	1	1
REAC:R-HSA-6798695	Neutrophil degranulation	-	1.42 × 10^−1^	1.96 × 10^−3^	-	1.63 × 10^−4^	2.40 × 10^−13^
REAC:R-HSA-909733	Interferon alpha/beta signaling	1.61 × 10^−8^	2.26 × 10^−6^	4.48 × 10^−4^	1	1	1
REAC:R-HSA-913531	Interferon Signaling	2.77 × 10^−8^	3.12 × 10^−7^	3.17 × 10^−9^	1	1	1

**Table 2:** Enrichment analysis of KEGG and Reactome pathways with gProfiler. Column ‘decreased’ shows results obtained with genes that were detected in mock-infected cells, but not after ASFV infection. Additionally, the genes present in the 10% percentile of the most strongly decreased synthesis rates are presented, and results from the ‘increased’ column include genes from the 10% percentile of the most strongly increased synthesis rates and those that were detected exclusively after ASFV infection. White—not significantly enriched; light grey—*p* < 0.05; dark grey—*p* < 0.01; black—*p* < 0.001.

Term ID	Term Name	Decreased	Increased
KEGG:01100	Metabolic pathways		
KEGG:04142	Lysosome		
KEGG:05022	Pathways of neurodegeneration—multiple diseases		
REAC:R-HSA-168249	Innate immune system		
REAC:R-HSA-168256	Immune system		
REAC:R-HSA-1169410	Antiviral mechanism by IFN-stimulated genes		
REAC:R-HSA-1169408	ISG15 antiviral mechanism		
REAC:R-HSA-1280215	Cytokine signaling in immune system		
REAC:R-HSA-913531	Interferon signaling		
REAC:R-HSA-909733	Interferon alpha/beta signaling		
REAC:R-HSA-199991 REAC:R-HSA-5653656	Membrane trafficking		
REAC:R-HSA-199977 REAC:R-HSA-6807878	ER to Golgi anterograde transport		
	
REAC:R-HSA-8953854 REAC:R-HSA-194441	Metabolism of RNA		
REAC:R-HSA-72203	Processing of capped intron-containing pre-mRNA		
REAC:R-HSA-72163 REAC:R-HSA-72172	mRNA splicing		

REAC:R-HSA-379724 REAC:R-HSA-379716	tRNA aminoacylation		
REAC:R-HSA-6784531	tRNA processing in the nucleus		
REAC:R-HSA-72202	Transport of mature transcript to cytoplasm		
KEGG:03013	Nucleocytoplasmic transport		
REAC:R-HSA-9615933 REAC:R-HSA-3301854	Nuclear Pore Complex (NPC) disassembly and reformation		
REAC:R-HSA-4085377 REAC:R-HSA-4615885 REAC:R-HSA-3232142	SUMOylation of proteins		
REAC:R-HSA-5213460 REAC:R-HSA-9686347	RIPK1-mediated regulated necrosis		
REAC:R-HSA-5675482 REAC:R-HSA-5218859	Regulation of necroptotic cell death		
KEGG:04210	Apoptosis		

## Data Availability

The mass spectrometry proteomics data have been deposited to the ProteomeXchange Consortium (http://proteomecentral.proteomexchange.org accessed on 2 February 2023) via the PRIDE partner repository [58] with the dataset identifier PXD039806.

## Supplementary Materials

The following supporting information can be downloaded at: https://www.mdpi.com/article/10.3390/v15061283/s1, Figure S1: Confirmation of ASFV and PrV infections; Figure S2: Correlation of relative synthesis rates and protein abundance levels in mock-infected and PrV-infected moMΦ; Table S1: Identifications, relative synthesis rates, and absolute quantifications of ASFV-specific, PrV-specific, and porcine proteins (Supplementary File); Table S2: Identifications and relative synthesis rates ASFV and PrV proteins (Supplementary File); Table S3: List of selected porcine genes with increased and decreased synthesis rates for enrichment analysis (Supplementary File); Table S4: Results of multiquery enrichment analysis of Reactome and KEGG-pathways (Supplementary File).
