# Supplementary material for: Mass-Spectrometric Evaluation of the African Swine Fever Virus-Induced Host Shutoff Using Dynamic Stable Isotope Labeling with Amino Acids in Cell Culture (SILAC)
Source: Viruses. 2023 May 30;15(6):1283. doi: 10.3390/v15061283 (PMC10305699; doi:10.3390/v15061283)
Supplement: Supplementary file 1 [file viruses-15-01283-s001.zip › EWoehnke_shutoff_supplement.pdf]

Supplementary Information

Title

Mass-spectrometric evaluation of the African swine fever virus-induced host shutoff using dynamic stable isotope labeling with amino acids in cell culture (SILAC)

Authors:

Elisabeth Wöhnke, Barbara G. Klupp, Sandra Blome, Thomas C. Mettenleiter and Axel Karger

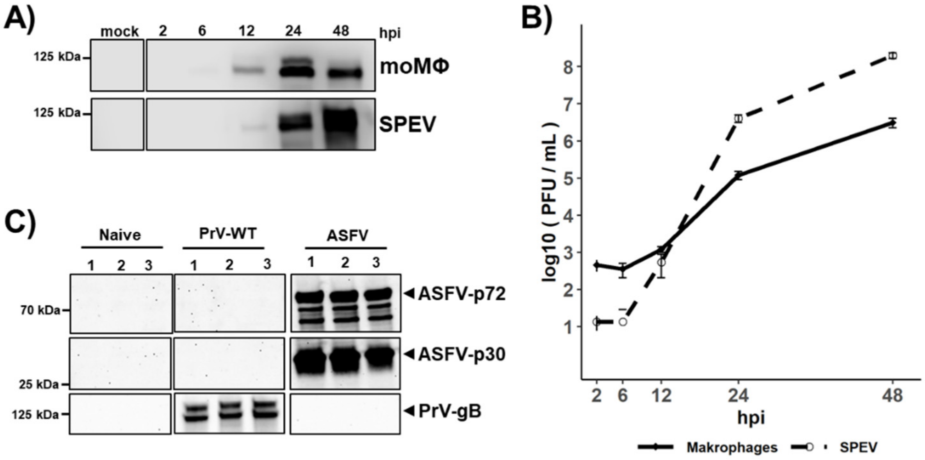

Figure S1: Confirmation of ASFV and PrV infections. Expression of PrV-gB (A) and PrV titers as PFU/mL (B) after infection of SPEV cells and moMΦ were monitored over 48h. PrV titers were determined by titration on MDBK cells. C) Before MS analysis, infections of moMΦ with PrV (16 hpi) and ASFV (24 hpi) were confirmed by detection of PrV-gB, ASFV-p72, and ASFV-p30 in Western Blots.

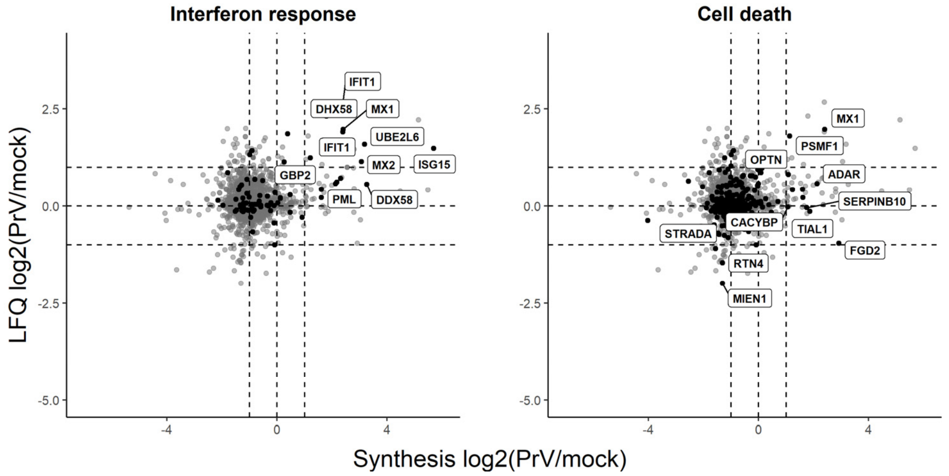

Figure S2: Correlation of relative synthesis rates and protein abundance levels in mock-infected and PrV-infected moMΦ. Relative protein synthesis rates (PrV-infected/mock-infected, based on the SILAC measurement) and relative protein abundance levels (PrV-infected/mock-infected, based on LFQ measurement) are presented as log2 values. Dotted horizontal and vertical lines indicate zero change and 2-fold changes in either direction. In the left and right panels genes related to the interferon response and to cell death are highlighted, respectively.

Table S1: Identification, relative synthesis rates, and absolute quantification of porcine proteins based on label-free quantification (LFQ) results from Fragpipe analysis. Identifications are based on annotated sequences from the host (S. scrofa; downloaded from Ensembl repository). Relative synthesis rates were defined as the ratio of newly synthesized protein over the pre-existing amount of protein and calculated based on the experimental heavy-to-light ratios. (supplementary file)

Table S2: Identification, heavy-to-light ratios (HoL), and relative synthesis rates of ASFV and PrV proteins based on Fragpipe analysis. Identification is based on annotated sequences of the virus proteomes (ASFV-Georgia (GenBank FR682468.2) or PrV-Kaplan (Genbank NC_006151). Relative synthesis rates were defined as the ratio of newly synthesized protein over the pre-existing amount of protein and calculated based on the experimental heavy-to-light ratios. (supplementary file)

Table S3: List of selected porcine genes with increased and decreased synthesis rates for enrichment analysis selected on basis of the 10% percentiles of synthesis rates and qualitative observations. (supplementary file)

Table S4: Results of a gProfiler multiquery enrichment analysis of Reactome and KEGG-pathways based on genes with increased or decreased synthesis rates (see Table S2). Term and group-specific p-values are shown rounded to 4 digits. (supplementary file)
